# The importance of ultrasound examination in care of juvenile idiopathic arthritis patients: 9 months follow-up study

**DOI:** 10.3389/fped.2024.1414384

**Published:** 2024-09-12

**Authors:** Ausra Snipaitiene, Andzelika Slegeryte, Rimantas Uktveris, Rima Sileikiene, Paulius Jakucionis, Asta Baranauskaite, Lina Jankauskaite

**Affiliations:** ^1^ Pediatric Department, Faculty of Medicine, Lithuanian University of Health Sciences, Kaunas, Lithuania; ^2^ Radiology Department, Faculty of Medicine, Lithuanian University of Health Sciences, Kaunas, Lithuania; ^3^ Rheumatology Department, Faculty of Medicine, Lithuanian University of Health Sciences, Kaunas, Lithuania; ^4^ Faculty of Medicine, Institute of Physiology and Pharmacology, Lithuanian University of Health Sciences, Kaunas, Lithuania

**Keywords:** juvenile idiopathic arthritis, MSUS, ultrasound, imaging, children, pediatric

## Abstract

**Introduction:**

Juvenile idiopathic arthritis (JIA) is a group of rare musculoskeletal disorders with chronic inflammation of joints, typically manifesting before the age of 16 years. The assessment of disease activity remains pivotal in JIA treatment decisions, particularly during clinical remission. While musculoskeletal ultrasound (MSUS) has shown promise in detecting subclinical synovitis, longitudinal data on MSUS features in JIA remains limited. The aim of this study was to evaluate the prevalence of subclinical synovitis observed in MSUS over a follow-up period in JIA patients. Additionally, it sought to assess the consistency and correlation between clinical findings, standardized composite clinical score (JADAS10), and MSUS-detected synovitis during 9 months follow-up.

**Patients and methods:**

a prospective single-center study was conducted, enrolling all consecutive JIA patients (excluding systemic JIA) seen at the study center in one year period. At three-months intervals over a 9 months period (M0, M3, M6 and M9), patients underwent clinical examination, laboratory tests, and MSUS assessment. Data on demographic characteristics, disease profile, and treatment were collected. Patients were categorized into active disease (ACT) or remission (REM) groups based on Wallace criteria and JADAS10 scores using previously validated thresholds. The ultrasound assessments adhered to the Outcome Measures in Rheumatology Clinical Trials (OMERACT) pediatric group, covering 40 joints, were performed by two ultrasonographers at every visit. Subclinical synovitis was defined as synovitis detected exclusively by MSUS. Spearman's correlation coefficients (r_s_) were used to evaluate the association between MSUS, clinical data, and outcome measures, such as active joint count (ACJ), patient's/parent's global assessment of disease activity (PaGA), physician's global assessment of disease activity (PhGA) and JADAS10.

**Results:**

subclinical synovitis was evident in 5.2% of all joints and in 80.6% of the patients at baseline. During the follow-up period, signs of subclinical synovitis decreased to 3.8% of joints, however, the proportion of affected patients remained high (67.7%), with the majority in REM group. Despite the consistent strong correlation between PaGA and PhGA throughout the study (r_s _> 0.895; *p* < 0.001), both measures displayed moderate (r_s _= 0.647; *p* < 0.001) to weak (r_s _= 0.377; *p* = 0.04) correlations with MSUS findings. Notably, PaGA remained significantly correlated with MSUS at the M9 visit (r_s _= 0.377, *p* = 0.04), while PhGA showed no correlation (*p* = 0.094).

**Conclusions:**

The study results indicate the persistence of subclinical inflammation detected by MSUS in a significant proportion of JIA patients, even during clinical remission. Moreover, the findings suggest that conventional measurements of JIA activity may be insufficient for assessing patients in clinical remission.

## 1 Introduction

Juvenile idiopathic arthritis (JIA) comprises a group of rare musculoskeletal disorders characterized by predominant chronic joint inflammation starting before the age of 16 (1). Recent studies have elucidated distinctions in pathogenesis, genetic predisposition and epidemiology among clinical JIA forms, such as oligoarthritis, polyarthritis, enthesitis-related arthritis, and others, varying across different regions (2–5). Assessment of disease activity remains pivotal in guiding treatment decisions for JIA. Currently, clinical assessment tools are widely utilized and validated for monitoring JIA activity in children (6, 7). Nonetheless, these tools encounter several limitations including various modifications developed for different JIA subtypes with diverse reference values for disease activity (7–10). Moreover, challenges also arise from factors such as young age (resulting in limited patient collaboration, age-related joint hypermobility, and an absence of self-reported joint pain) as well as transient childhood joint disorders (e.g., transient synovitis). Additionally, studies have revealed poor-to-moderate interrater agreement in the clinical arthritis assessment, along with various factors influencing physicians’ evaluation of global JIA disease activity (11, 12). Consequently, pediatric rheumatologists are actively seeking more reliable and objective tools or biomarkers for assessing JIA activity in pediatric patients. Due to this, in recent years, musculoskeletal ultrasound (MSUS) has gained increasing interest among pediatric rheumatologists for evaluating chronic inflammation. The non-invasive, radiation-free, inexpensive, and highly patient-friendly nature of MSUS can help to delineate the extent of joint involvement in JIA.

Several previous studies on JIA patients have underscored the importance of detecting subclinical synovitis using MSUS for defining JIA subtypes and assessing active joint count (13–15). Recent efforts have focused on standardizing scanning protocols and synovitis evaluation across different joints in both JIA patients and healthy children, conducted by various study groups (16–19). However, longitudinal data on MSUS features in JIA patients throughout the disease course remain limited.

The aim of this study was to assess the frequency of subclinical synovitis observed via MSUS over a nine-month follow-up period in JIA patients. Additionally, it aimed to evaluate the consistency and correlation between clinical findings, standardized composite clinical score, and MSUS-detected during 9 months follow-up.

## 2 Materials and methods

### 2.1 Study design and study population

A prospective single-center study was conducted at the Hospital of Lithuanian University of Health Sciences enrolling all consecutive patients diagnosed with JIA (excluding systemic JIA) from January 2021 to March 2023. Inclusion criteria comprised: (1) diagnosis of JIA according to the International League of Associations for Rheumatology (ILAR) criteria (20); (2) age between 2 and 18 years. Exclusion criteria included: (1) systemic JIA form; (2) comorbid chronic diseases. Patients underwent regular follow-ups every three months for a nine-months period (M0, M3, M6 and M9), involving clinical examinations, laboratory tests, and MSUS assessments at each visit.

### 2.2 Data collection

Demographic data, including age and gender, disease characteristics, and treatment, were collected. Disease characteristic data included disease subtype according to ILAR categories, disease duration, presence/absence of rheumatoid factor (RF), antinuclear antibodies (ANA), and human leucocyte antigen B27 (HLA B27) results. The latest ophthalmological examination findings for signs of uveitis were reviewed. Moreover, signs of joint inflammation, such as swelling, pain, limited range of motion (LOM), and morning stiffness, were evaluated. All clinical signs were assigned a score of 0 (absent) or 1 (present) and quantified as the sum variable (ClinSUM) (total score range 0–8) for each patient for subsequent analysis. Each variable was considered with the same weight (present or absent) trying to minimize the redundant scoring of severity (21). Clinical evaluation was performed by a pediatric rheumatologist (ASn) certified in joint examination by the Pediatric International Trials Organisation (PRINTO). JIA disease activity was assessed using the validated clinical juvenile arthritis disease activity scale of 10 joints (JADAS10) (22). Additionally, clinical outcome measures, such as active joint count (AJC), defined as a joint with presence of swelling or, if no swelling was present, of pain on motion, or limited range of motion, patient global assessment of disease activity (PaGA) using visual analogue scale of 10 (where 0 = no activity and 10 = maximum activity), and physician global assessment of disease activity (PhGA) using visual analogue scale of 10 (where 0 = no activity and 10 = maximum activity), were evaluated.

Inflammation markers including erythrocyte sedimentation rate (ESR) and C-reactive protein (CRP) were assessed at each visit. Data about medications used for JIA treatment were also collected.

Patients were categorized into an active disease (ACT) or remission (REM) groups based on the Wallace criteria (23) and JADAS10 scale using previously validated cutoffs (7).

### 2.3 Ultrasound assessment

At each visit, MSUS of 40 joints was performed by two proficient ultrasonographers: a radiologist with over 30 years of expertise in pediatric MSUS (RU), blinded to clinical examination, and a pediatric rheumatologist (ASn), who completed the European Alliance of Associations for Rheumatology (EULAR) intermediate course of pediatric MSUS and has six years of daily practice with pediatric patients. Gray scale (B-mode) and power Doppler (PD) ultrasonographic evaluations were performed using an Affinity 70G (Philips) and ACUSON Sequoia™ (Siemens Healthineers) machines with a linear-transducers with frequency range from 5 to 18 Mega Hertz (MHz) for grayscale and up to 12.5 MHz for PD. A pulse repetition frequency of 500–900 MHz with a low-wall filter was used, adjusting the gain to eliminate signals on or below the bone surface. B-mode and Power Doppler images were acquired and scored from 0 to 3 for each of the 40 joints following the guidelines outlined by the Outcome Measures in Rheumatology Clinical Trials (OMERACT) group (17, 24, 25). Subclinical synovitis was defined as synovitis detected exclusively by MSUS.

In total, 1,240 joints were assessed using ultrasound every visit by two examiners. For analysis joints were grouped as follows: (1) small hand joints (SHJ), comprising MCP (metacarpophalangeal) and PIP (proximal interphalangeal) joints; (2) ankle (tibiotalar, talonavicular, subtalar joints); (3) all MTPs (metatarsophalangeal joints). Hips, knees, wrists, and elbows were rated separately.

Inter-rater reliability, particularly concerning the identification of grey scale or Doppler abnormalities, was highest in elbows, wrists and hips (kappa = 1), with the greatest disparity observed in MTP joints (kappa 0.890) (Supplementary Table S1).

### 2.4 Outcome and MSUS statistical analysis

Statistical analysis was performed using Microsoft Excel and IBM SPSS Statistics version 29.0 software (SPSS Inc., Chicago, IL, USA) for Windows. The Shapiro-Wilk test was used to assess the data normality. Continuous variables were presented as mean ± standard deviation (SD) or median and interquartile range (IQR). Qualitative data were expressed as counts and percentages (%).

The independent samples *t*-test was applied for normally distributed data to compare different follow-up visits, whereas the Wilcoxon signed ranks and Mann-Whitney *U*-tests analysed nonparametric data. Inter-rater reliability was evaluated using Cohen's kappa value, with cut-offs of below 0.20 indicating poor reliability, 0.21–0.40 indicating fair reliability, 0.41–0.60 indicating moderate reliability, 0.61–0.80 indicating good reliability, and 0.81–1 indicating excellent reliability. Spearman's correlation coefficients (r_s_) assessed associations between presence/absence of subclinical synovitis signs in MSUS at the patient level, clinical data, and outcome measures. The correlation between the number of patients with clinical symptoms and the number of patients with signs of synovitis in MSUS was performed. A *p*-value < 0.05 was considered significant. No correlated variables were marked as NC in the tables.

### 2.5 Ethical consent

Permission to conduct the study was granted by Kaunas Regional Biomedical Research Ethics Committee (BE-2-100; 2020 Oct 26). Study was conducted in accordance with the Declaration of Helsinki and Good Clinical Practice Guidelines.

## 3 Results

### 3.1 Patient characteristics and disease presentation

A total of 31 patients with JIA were included in the study, with a median age of 13.61 years (range 3–17 years). 87.1% of patients (27/31) were female. The median duration of JIA at the baseline visit was 21.6 months (min 0, max 98.7)). Almost half of the patients (45.2%) had the disease for more than one year, while only 9 patients (29%) had been ill less than 6 months (Table 1). According to ILAR JIA classification (20), 12 patients (38.7%) had oligoarthritis (extended or persistent), 10 patients (32.3%) RF negative polyarthritis, 1 patient (3.2%) RF positive polyarthritis, and 8 patients (25.8%) enthesitis-related arthritis. Antinuclear antibodies (ANA) were detected in two-thirds of the patients, and human leucocyte antigen B27 (HLA B27) in one-third of the patients (Table 1).

**Table 1:** Demographic and clinical features of the study cohort at baseline visit.

Characteristic		Total (*n* = 31)
Gender (n,%)	Female	27 (87.1)
	Male	4 (12.9)
Age at M0 in years, mean (SD)		13.61 (4.31)
Age of JIA diagnosis in years, mean (SD)		11.81 (4.74)
Disease duration in months, median (IQR)		10 (0–17.7)
Disease duration <6 months (*n*,%)		9 (29)
Disease duration from 6 months till 1year (*n*,%)		8 (25.8)
Disease duration >1 year (*n*,%)		14 (45.2)
Oligoarthritis (*n*,%)		12 (38.7)
RF negative polyarthritis (*n*,%)		10 (32.3)
RF positive polyarthritis (*n*,%)		1 (3.2)
Enthesitis related arthritis (*n*,%)		8 (25.8)
ANA positive (*n*,%)		19 (61.3)
HLA B27 positive (*n*,%)		10 (32.3)

ANA, antinuclear antibodies; AntiTNF, tumor necrosis factor inhibitors; HLA B27, human leucocyte antigen B27; n, number of patients; RF, rheumatoid factor; SD, standard deviation.

### 3.2 Clinical presentation and disease course

Regarding clinical arthritis presentation signs at the baseline visit, joint pain was reported by 67.7% of patients, while half of the children (54.8%) expressed complaints of joint swelling and LOM. Morning stiffness was present in only 6 patients (19.4%), and none of the children displayed signs of uveitis (Table 2). During the follow-up period, the majority of the symptoms resolved, with only 22.5% of patients experiencing joint pain, and joint swelling decreased to less than 20% (Table 2). Moreover, LOM and morning stiffness had completely disappeared.

**Table 2:** Frequency of patients with pathological clinical findings, according to disease activity status, outcome measures and treatment at baseline and at the last visit.

Characteristic	M0 visit (*n* = 31)	M9 visit (*n* = 31)
Clinical features		
Joint swelling (*n*,%)	17 (54.8)	6 (19.4)
Joint pain (*n*,%)	21 (67.7)	7 (22.5)
Limited range of motion (*n*,%)	17 (54.8)	0
Morning stiffness (*n*,%)	6 (19.4)	0
Uveitis (*n*,%)	0	0
Disease activity (DA)		
Remission (*n*,%)	8 (25.8)	25 (80.6)
Low DA (*n*,%)	3 (9.7)	5 (16.1)
Moderate DA (*n*,%)	4 (12.9)	0
High DA (*n*,%)	16 (51.6)	1 (3.2)
JADAS10 median (IQR)	7 (1–12)	0 (0–1)*
PhGA median (IQR)	3 (1–5)	0 (0–0.5)*
PaGA median (IQR)	3 (0–5)	0 (0–1)*
ClinSUM median (IQR)	2 (0–3)	0 (0–1)
Treatment		
• None (*n*,%)	2 (6.7)	5 (16.1)
• NSAIDs (*n*,%)	13 (41.9)	1 (3.2)
• Prednisone (*n*,%)	1 (3.2)	0
• Intraarticular injections of steroids (*n*,%)	2 (6.5)	0
• MTX (*n*,%)	22 (71)	22 (71)
• Sulfa (*n*,%)	2 (6.5)	1 (3.2)
AntiTNF (*n*,%)	11 (35.5)	16 (51.6)
MTX + antiTNF (*n*,%)	7 (22.6)	12 (38.7)

AntiTNF, tumor necrosis factor inhibitors; DA, disease activity; IQR, interquartile range; JADAS10, juvenile arthritis disease activity score 10; PhGA, physician global assessment of DA; PaGA, patient/parent global assessment of DA; M0, baseline visit; M9, nine months from inclusion into the study; MTX, methotrexate; n, number; NSAIDs, non-steroidal anti-inflammatory drugs; Sulfa, sulfasalazine. Statistically significant differences between visits are marked with *. *P* < 0.05 was considered significant.

### 3.3 Disease activity

According to JADAS10 cut-offs, half of the cohort (51.6%) was categorized having high disease activity during the baseline visit (Table 2). Only 8 patients (25.8%) achieved clinical remission according to both JADAS10 and Wallace criteria (23) for more than 6 months. Notably, patients with shorter disease duration at the M0 visit had higher disease activity (Table 3). By the M9 visit the remission group significantly increased to 80.6%, with 5 patients exhibiting low disease activity according to JADAS10, and only one patient was classified as having high JIA activity (Table 2). The median JADAS10 score between the M0 visit and M9 visit decreased from 7 to 0 (*p* < 0.001). Furthermore, the outcome measures such as PhGA and PaGA also decreased significantly during the 9 months follow-up period (Table 2).

**Table 3:** Disease activity according to disease duration at baseline visit.

Total, *n* = 31	Disease duration		
Disease activity	Up to 6 mo	6mo—1y	More than 1y
High, *n* (%)	9 (29)	6 (19.4)	1 (3.2)
Moderate, *n* (%)	0	1 (3.2)	3 (9.7)
Low, *n* (%)	0	1 (3.2)	2 (6.5)
Inactive/REM, *n* (%)	0	0	8 (25.8)

y, years; mo, months; n, number; REM, remission.

Regarding conventional inflammation markers such as ESR and CRP, only 4 patients (12.9%) exhibited an elevation in CRP during the initial visit, which normalized in subsequent visits. There were no significant changes in ESR during all follow-up visits.

### 3.4 Treatment

The majority of the patients (71%) was undergoing treatment with methotrexate (MTX), 35.5% were prescribed tumor necrosis factor inhibitors (anti-TNF) at the time of inclusion in the study. 22.6% of patients were receiving combined therapy with MTX and anti-TNF. Additionally, 41.9% of patients were using nonsteroidal anti-inflammatory drugs (NSAIDs). Despite the treatments, 74.2% of children still exhibited some degree of disease activity during the baseline visit. By the M9 visit, the prescription of anti-TNF increased to 51.6%, and NSAIDs consumption decreased to 3.2%, resulting in the significant increase of REM group (Table 2).

### 3.5 Longitudinal assessment of clinical outcome measures and subclinical synovitis in MSUS

Several clinical outcome measures were evaluated at each visit for all patients. A notable positive correlation was found between the cumulative value of all clinical signs (ClinSUM) with MSUS-detected synovitis at M0, M3, and M6 visits (M0 r_s _= 0.678; M3 r_s _= 0.736; M6 r_s _= 0.716; *p* < 0.001). However, at the M9 visit, when the majority of the patients were in clinical remission for more than 6 months, the correlation weakened (r_s _= 0.309; *p* < 0.097). A similar trend was identified between JADAS10, AJC and MSUS examination results (Table 4).

**Table 4:** Correlation of clinical symptoms, inflammation markers and patient- and physician-reported measures with MSUS synovitis.

	MSUS synovitis			
	M0 r_s_	M3 r_s_	M6 r_s_	M9 r_s_
ClinSUM	0.678*	0.736*	0.716*	0.309
Joint Pain	0.757*	0.557*	0.559*	0.142
Joint Swelling	0.477*	0.753*	0.667*	0.130
LOM	0.046	0.200	NC	NC
AJC	0.701*	0.720*	0.741*	0.386*
JADAS10	0.69*	0.617*	0.685*	0.354
ESR	−0.007	−0.199	−0.332	−0.170
CRP	0.336	0.638*	−0.119	−0.082
PhGA	0.597*	0.484*	0.647*	0.311
PaGA	0.562*	0.474*	0.588*	0.377*

The correlation between the number of patients with clinical symptoms and the number of patients with signs of synovitis in MSUS was performed. The absolute ESR and CRP values for correlation with synovitis in MSUS were used.

ACJ, active joint count; ClinSUM, sum of all clinical signs; cJADAS, clinical juvenile arthritis disease activity scale; ESR, erythrocyte sedimentation rate; CRP, C reactive protein; LOM, limited range of motion; M0, baseline visit, M3, 3 months from inclusion into the study; M6, 6 months from inclusion into the study; M9, 9 months from inclusion into the study; NC, non correlated as there were no LOM registered for any patient; PaGA, patient/parent global disease activity; PhGA, physician's global disease activity; r_s_, Pearson's correlation coefficient. *P* < 0.05 was considered significant. Statistically significant results are provided with*.

Analysis of separate arthritis symptoms, such as joint pain and swelling, also resulted in significant positive correlations with MSUS in the first 3 visits (pain and MSUS: M0 r_s _= 0.757; M3 r_s _= 0.557; M6 r_s _= 0.559; *p* < 0.01; swelling and MSUS: M0 r_s _= 0.477; M3 r_s _= 0.753; M6 r_s _= 0.667; *p* < 0.001) and with no correlation at the M9 visit (*p* = 0.453, *p* = 0.492, respectively for pain and swelling) (Table 4). Interestingly, LOM showed no correlation with MSUS in any visit (Table 4).

Moreover, there was no association seen between MSUS and blood inflammation markers (ESR and CRP) throughout all follow-up visits (Table 4).

Despite the consistently strong correlation between PaGA and PhGA throughout the follow-up period (r_s _> 0.895; *p* < 0.001), both outcome measures displayed moderate (r_s _= 0.647; *p* < 0.001) to weak (r_s _= 0.377; *p* = 0.04) correlations with MSUS (Table 4). Notably, the correlation of PaGA with MSUS remained statistically significant at the M9 visit (r_s _= 0.377, *p* = 0.04), while PhGA showed no correlation (*p* = 0.094).

Subclinical synovitis was evident in 5.2% of all joints (Table 5) and in the majority of patients (80.6%) at the M0 visit (Table 6). During the follow-up period, the total number of joints with subclinical synovitis decreased to 3.8%; however, the proportion of affected patients remained high (67.7%), with the majority being in the REM group (Table 6). The joints most affected by subclinical synovitis on MSUS at the M0 visit were knees (16.7% of all knee joints) and ankles (16.7%), followed by subtalar joint (8.3%), elbows (6.7%), wrists (6.7%), proximal interphalangeal joints (PIP) (4.3%), MTPs (3.3%), and MCPs (1.3%) (Table 5). At the M9 visit, subclinical synovitis remained mostly in knees (18.3%), ankles (11.7%), elbows (13.3%), and wrists (8.3%).

**Table 5:** Clinical and MSUS signs of inflammation in different joints at baseline visit and at the last visit.

Joints examined in total (n)	M0 visit				M9 visit			
	Joints with arthritis by physical examination only (%)	Joints with arthritis by MSUS only (%)	Joints with arthritis on both physical examination and MSUS (%)	Total joints affected (%)	Joints with arthritis by physical examination only (%)	Joints with arthritis by MSUS only (%)	Joints with arthritis on both physical examination and MSUS (%)	Total joints affected (%)
Elbows (*n* = 60)	1 (1.7)	4 (6.7)	0	5 (8.3)	0	8 (13.3)	0	8 (13.3)
Wrists (*n* = 60)	1 (1.7)	4 (6.7)	3 (5)	8 (13.3)	0	5 (8.3)	3 (5)	8 (13.3)
MCPs (*n* = 300)	1 (0.3)	4 (1.3)	1 (0.3)	6 (2)	0	4 (1.3)	0	4 (1.3)
PIPs (*n* = 300)	0	13 (4.3)	11 (3.7)	24 (8)	6 (2)	2 (0.7)	1 (0.3)	9 (3)
Hips (*n* = 60)	3 (5)	0	1 (1.7)	4 (6.7)	0	1 (1.7)	0	1 (1.7)
Knees (*n* = 60)	1 (1.7)	10 (16.7)	10 (16.7)	21 (35)	0	11 (18.3)	4 (6.7)	15 (25)
Ankles (*n* = 60)	4 (6.7)	10 (16.7)	2 (3.3)	16 (26.7)	2 (3.3)	7 (11.7)	1 (1.7)	10 (16.7)
Subtalar (*n* = 60)	0	5 (8.3)	0	5 (8.3)	0	3 (5)	0	3 (5)
MTPs (*n* = 300)	3 (1)	10 (3.3)	3 (1)	16 (5.3)	0	6 (2)	1 (0.3)	7 (2.3)
Total (*n* = 1,240)	14 (5.8)	64 (5.2)	31 (2.5)	105 (8.5)	8 (0.6)	47 (3.8)	10 (0.8)	65 (5.2)

MSUS, musculoskeletal ultrasound; MCPs, metacarpophalangeal joints; PIPs, proximal interphalangeal joints; MTPs, metatarsophalangeal joints; M0, baseline visit; M9, nine-month visit from inclusion into the study.

**Table 6:** Patient proportion in different visits according to disease activity and MSUS synovitis.

Disease activity	M0 visit MSUS synovitis, *n* (%)	M3 visit MSUS synovitis, *n* (%)	M6 visit MSUS synovitis, *n* (%)	M9 visit MSUS synovitis, *n* (%)
High	13 (41.9)	8 (25.8)	5 (16.1)	1 (3.2)
Moderate	4 (12.9)	5 (16.1)	6 (19.4)	2 (6.5)
Low	1 (3.2)	4 (12.9)	1 (3.2)	4 (12.9)
Inactive/REM	7 (22.6)	7 (22.6)	5 (16.1)	14 (45.2)
Total	25 (80.6)	24 (77.4)	17 (54.8)	21 (67.7)

MSUS, musculoskeletal ultrasound; M0, baseline visit; M3, 3 months from inclusion into the study; M6, 6 months from inclusion into the study; M9, nine-month visit from inclusion into the study.

Furthermore, the correlation between clinical examination and MSUS of different joints did not show consistent pattern. Correlation could not be calculated for some of the joints due to the absence of clinical signs of arthritis (Supplementary Table S2).

## 4 Discussion

Juvenile idiopathic arthritis (JIA), although rare, ranks among the most prevalent chronic rheumatological conditions affecting children (2, 26). Regular assessment of disease activity and potential treatment adjustments are necessary in any type of JIA. While various clinical tools are employed to evaluate JIA activity (7), an increasing body of evidence suggest implication of ultrasound (13, 27–30). In our prospective study with a 9-month follow-up, disease activity in 31 JIA patients was assessed through both clinical examination and MSUS. Several clinical outcome measures demonstrated a strong correlation with MSUS during the initial 6 months of the disease. However, at the 9-months mark, the majority of patients continued to display signs of inflammation in MSUS despite achieving clinical remission.

Subclinical inflammation signs in MSUS among JIA patients have been recognized for several years (14, 27–29, 31, 32). However, most studies have focused on newly diagnosed or short-term remission JIA cases (13, 14, 32), and the majority did not utilize validated MSUS definitions for pediatric population. In our study, we applied the OMERACT group definitions and criteria for MSUS in pediatric patients (17, 24, 25). Only a quarter of our study patients at the baseline visit could be classified as in clinical remission according to the Wallace criteria (23) and as having inactive disease according to JADAS10 cut-offs (7). Naturally, more patients with longer disease duration were classified as inactive. However, the majority still exhibited signs of synovitis in MSUS. Similar results were described in a few previous studies. In De Lucia et al.'s study, 22.7% of clinically inactive patients and 0.98% of scanned joints showed abnormal MSUS results (33). Loredo et al. observed 11.8% of JIA patients in remission displaying subclinical inflammation in MSUS over a year (34). Bugni Miotto e Silva at al. found a higher prevalence of subclinical synovitis in 36 JIA patients (median duration: 1.9 years), with 41.7% of patients and 3.1% of joints affected (29).

The relationship between clinically active JIA and inflammation signs in ultrasound is well-established. However, considerable portion of active JIA patients also demonstrated signs of subclinical synovitis in MSUS. In a recent study by Vega-Fernandez et al. up to 30% of joints of clinically active patients had signs of subclinical inflammation on MSUS (13). Several other studies reported a lower number of subclinical synovitis cases in active JIA group (32, 34, 35). The correlation of ACJ with MSUS signs of inflammation in active or newly diagnosed JIA is well-known and robust (34–36). In our patient cohort, half exhibited high disease activity according to JADAS10 cut-offs at the baseline visit, and most of them had signs of subclinical synovitis on MSUS. Moreover, inflammation signs on MSUS were observed in most patients classified as having JIA remission. Nevertheless, our study revealed a strong positive relationship between ACJ and MSUS at baseline and in earlier follow-up visits (M3 and M6). However, during the remission phase in the last visit (M9), this correlation weakened, indicating persistent sings of synovitis in MSUS. Similar findings were found in recent study by Vega-Fernandez et al. which evaluated active JIA patients after 3 months of follow-up (13).

The distribution of inflammation signs variations among the different joints has been noted in several previous multi-joint scanning studies (29, 31, 32, 35). The joints most prone to subclinical inflammation signs on MSUS are knees, ankles and wrists (29, 31, 32, 35). The same observation was made in our scanned joints complex, with knees and ankles standing out throughout all follow-up period. Moreover, the MSUS correlation with clinical signs in the knee joint was significantly moderate in baseline visit and 3 months after (r*s* = 0.52, *p* = 0.003 and r*s* = 0.45, *p* = 0.012, respectively), appending previous results by several studies (35, 37).

Clinical signs of arthritis serve as the core measures in JIA diagnosis, definition of JIA category and disease activity evaluation. From the first studies on MSUS in JIA patients, it has been noted that different clinical signs have different correlations with MSUS. Magni-Manzoni et al. described poor correlation of MSUS with tenderness, pain on joint movement, or LOM. Only swelling showed a moderate to strong correlation with MSUS (14). In our study clinical signs like joint pain and swelling showed moderate to strong correlation with MSUS during the first 6 months of follow-up. Interestingly, we did not find any correlation of MSUS with LOM in any visit, emphasizing the importance of composite clinical evaluation of the patient.

Currently, the measurement of JIA disease activity involves several clinical outcome measures, some of which are integrated into joint tools such as various modifications of JADAS (cJADAS, JADAS10, JADAS27 etc.) (7). Several research groups have identified a significant correlation between JADAS and MSUS in active JIA patients (35). In our study, we also observed a significant positive moderate correlation between JADAS10 and inflammation signs in MSUS during the initial 6 months of the follow-up. However, no correlation was found at the M9 visit, when the majority of patients were in clinical remission, mainly due to persisting signs of synovitis in MSUS. Moreover, a recent study by Licciardi et al. described a significant correlation of JADAS27 with MSUS in the active JIA group but not in remission (35).

Conflicting results regarding the correlation between other clinical disease activity measurements, such as physician's and patient's/parent's global disease activity evaluation and MSUS have been observed in several studies. Bugni Miotto e Silva et al. analysed patients with a median remission time of 1.9 years and did not find relationship between MSUS detectable synovitis and clinical disease activity evaluation by PhGA or PaGA (29). Moreover, a recent study by Nguyen et al. underscores that clinical outcome measures do not uniformly change during the disease course (38). Despite an increased proportion of inactive JIA patients according to cJADAS and PhGA, little or no improvement was seen in PaGA of JIA activity (38). Interestingly, in our cohort of patients, the correlation between PaGA and MSUS remained positive at 9 months of follow-up despite clinical signs of inflammation disappearing, and no relationship was found between MSUS and PhGA. These findings suggest that the perception of disease activity by patients or parents may be more sensitive to subclinical inflammation within the body. A similar tendency of consistently elevated PaGA scores was evidenced in a prospective multicenter study encompassing over 1,000 JIA patients (39). Researchers identified older age and enthesitis-related JIA as potential risk factors for prolonged elevation of PaGA. Moreover, a longitudinal, population-based study conducted by Rypdal et al. delineated PaGA as a principal factor associated with higher JADAS10 values (40). Our study indicated that subclinical synovitis detected via MSUS could be one of the explanations for prolonged elevation of PaGA.

Our study has some limitations. Firstly, a small number of JIA patients was evaluated. However, given that JIA is a rare disease and considering the 9-months follow-up period, the study population is similar to the cohorts of some previous single-center studies (13, 29–31, 35, 41). A second limitation is the variety of JIA subgroups included, making the cohort less homogenous. However, previously validated clinical disease activity tool JADAS10 and separate cut-offs of this scale for oligo and polyarticular JIA were used to classify patients according to the disease activity level. This approach ensures that the full spectrum of non-systemic JIA manifestations included provides a wide view of the MSUS synovitis signs in different JIA groups in remission. Moreover, the evaluation of a variety of peripheral joints by MSUS (1,240 joints at each visit) contributes to some of the largest studies in pediatric population done so far (13, 28, 29, 33).

Strengths of this study include the evaluation of joints according to criteria tailored specifically for pediatric patients. Additionally, regular follow-up visits every 3 months throughout the 9-months period were conducted, whereas most studies only included two visits at different time points or one visit across different JIA activity groups (13, 28, 29, 34). Furthermore, the parallel evaluation of all children in all visits by two ultrasonographers on the same day in real-time adds an additional advantage in inter-rater reliability. Our study demonstrated that despite different specialties and experience in ultrasound, MSUS is a reliable imaging modality for evaluating inflammation in JIA patients when appropriate guidelines are followed.

Currently, the definition of remission or an inactive state of JIA does not include imaging results (7, 23). However, as more data emerges on the importance of MSUS, ultrasound findings should be considered alongside clinical assessments for a comprehensive evaluation of disease activity and could potentially be included as an additional criterion for defining JIA remission in the future.

In conclusion, our study indicates that despite being in clinical remission for 6 months or more, a significant number of JIA patients exhibit signs of subclinical inflammation in MSUS. The study highlights the importance of MSUS in assessing JIA patients in remission. Overall, ultrasound findings provide a valuable insight into disease activity and should be integrated into routine clinical practice for assessing and managing JIA patients.

## Data Availability

The raw data supporting the conclusions of this article will be made available by the authors, without undue reservation.
