# Supplementary material for: The importance of ultrasound examination in care of juvenile idiopathic arthritis patients: 9 months follow-up study
Source: Front Pediatr. 2024 Sep 12;12:1414384. doi: 10.3389/fped.2024.1414384 (PMC11424401; doi:10.3389/fped.2024.1414384)
Supplement: Supplementary file 1 [file Table1.pdf]

Supplementary Material

Supplementary Table 1. Inter-rater reliability for different joints.

Area	Total number	kappa value
**Elbows**	31	1
**Wrists**	31	1
**Small hand**	31	0.918
**Hips**	31	1
**Knees**	31	0.926
**Ankles**	31	0.912
**MTPs**	31	0.890

Abbreviations: MTPs – metatarsophalangeal joints.

Supplementary Table 2: Correlation between the presence of MSUS synovitis and the presence of clinical synovitis signs for different joints during the study period

Joints	M0 visit		M3 visit		M6 visit		M9 visit	
	r_s	p value	r_s	p value	r_s	p value	r_s	p value
**Elbows**	-0.062	0.745	NC	-	NC	-	NC	-
**Wrists**	0.361	0.050	0.230	0.222	0.447	0.013	NC	-
**MCPs**	0.408	0.025	0.650	<0.001	0.286	0.125	NC	-
**PIPs**	0.847	<0.001	-0.043	0.823	0.833	<0.001	0.557	0.001
**Hips**	0.274	0.142	NC	-	NC	-	NC	-
**Knees**	0.520	0.003	0.451	0.012	0.354	0.055	0.515	0.004

**Ankles**	0.098	0.608	0.280	0.134	0.604	<0.001	0.079	0.679
**Subtalar**	NC	-	NC	-	NC	-	NC	-
**MTPs**	0.347	0.061	0.088	0.645	0.282	0.130	0.777	<0.001

Abbreviations: MSUS – musculoskeletal ultrasound; MCPs – metacarpophalangeal joints; PIPs – proximal interphalangeal joints; MTPs – metatarsophalangeal joints; M0 – baseline visit; M3 – 3 months from inclusion into the study; M6 – 6 months from inclusion into the study; M9 – nine-month visit from inclusion into the study; NC – non correlated as there were no clinical signs detected.
